# Comparison of different 2D muscle indexes measured at the level of the 3rd lumbar vertebra in survival prediction in patients with renal cell carcinoma

**DOI:** 10.2340/1651-226X.2024.27450

**Published:** 2024-05-14

**Authors:** Oona Janhunen, Otto Jokelainen, Robin Peltoniemi, Timo K. Nykopp, Otso Arponen

**Affiliations:** a Institute of Clinical Medicine, University of Eastern Finland, Kuopio, Finland; b Clinical Pathology and Forensic Medicine, Institute of Clinical Medicine, School of Medicine, University of Eastern Finland, Kuopio, Finland; c Department of Clinical Pathology, Diagnostic Imaging Center, Kuopio University Hospital, Kuopio, Finland; d Faculty of Medicine and Health Technology, Tampere University, Tampere, Finland; e Department of Radiology, Tampere University Hospital, Tampere, Finland; f School of Medicine, Surgery, Institute of Clinical Medicine, University of Eastern Finland, Kuopio, Finland; g Division of Surgery, Department of Urology, Kuopio University Hospital, Kuopio, Finland; h Faculty of Medicine and Health Technology, Tampere University, Tampere, Finland; i Department of Oncology, Tays Cancer Centre, Tampere University Hospital, Tampere, Finland

**Keywords:** Renal cell cancer, sarcopenia, muscle index, body composition

## Abstract

**Background:**

Low computed tomography (CT)-determined muscle mass, commonly determined with height-adjusted muscle indexes (MIs), predicts worse survival in several cancers and has been suggested as a prognostic assessment tool. Although several MIs measured at the level of the 3rd lumbar vertebra (L3) are commonly used, it remains unestablished how different L3-determined MIs perform in survival prognostication compared to each other. The objective of this study was to investigate the performance of different MIs for survival prognostication in renal cell carcinoma (RCC).

**Methods:**

We retrospectively enrolled 214 consecutive patients with RCC. We determined three L3-MIs (psoas muscle index (PMI), psoas muscle index and erector spinae index (PMI+ESI), and whole skeletal muscle index (SMI)) from preoperative CT scans. Categorization of those with low and normal muscle mass was based on the Youden Index sex-specific MI cut-offs. We determined sensitivity, specificity, and accuracy metrics for predicting 1-year, 5-year, and overall survival (OS) using Cox regression models.

**Results:**

Low PMI, PMI+ESI, and SMI significantly predicted decreased 1-year, 5-year, and OS in uni- and multivariate models. PMI+ESI and SMI were more accurate than PMI in males, and PMI and PMI+ESI were more accurate than SMI in females in the prediction of 1-year survival. However, there were no differences in accuracies between MIs in 5-year and OS prediction.

**Interpretation:**

PMI+ESI performed well overall in short-term prognostication, but there were no differences between the MIs in long-term prognostication. We recommend the use of PMI+ESI for muscle evaluation, particularly when SMI cannot be evaluated.

## Introduction

Renal cell carcinoma (RCC) accounts for approximately >90% of kidney cancers and 2.4% of all cancers worldwide [1, 2]. RCC is the most lethal urological cancer [3]. Its incidence is the highest in developed countries in Europe and North America and is predicted to increase in the future as the Western lifestyle spreads and the population ages [2–4].

Muscle loss is an age-related process [5]. In addition to aging, low muscle mass and a loss of strength and muscle function characterizes sarcopenia [6, 7]. Loss of muscle mass and strength are also encountered in cachexia, malnutrition, and frailty [8]. Low muscle mass and sarcopenia have been associated with reduced survival [9, 10], quality of life [11], worse clinical outcomes (e.g., postoperative infections and surgical complications) [12], and greater toxicity of chemotherapy treatments [13] in patients with cancer. A recent systematic review employing computed tomography (CT)-determined muscle indexes (MIs) suggested that the median prevalence of low muscle mass is 43% in patients with cancer in general [14]. Similarly, low muscle mass has been suggested to range between 43%–44% in patients with RCC [15, 16]. Indeed, similar to many cancers [10, 17], low skeletal muscle mass (SMM) has been found to be an independent predictor of worse overall survival (OS) in both local [18–20] and metastatic RCC (mRCC) [21–23]. Low muscle mass is also associated with a risk of major complications after radical nephrectomy [24] and is a significant predictor of the toxicity of oncological treatments in mRCC [25, 26].

Imaging-determined evaluation of muscle mass based on height-adjusted MIs particularly at the level of the 3rd lumbar vertebra (L3) is of considerable interest as patients often undergo abdominal imaging [10, 27, 28]. As CT imaging is used to preoperatively stage and subsequently follow up with patients with RCC [29], the determination of MIs is possible at multiple time points before the possible surgical operation and during the course of systemic treatments in patients with mRCC. Several L3-MIs, such as the psoas muscle index (PMI), psoas muscle index and erector spinae (PMI+ESI), and whole skeletal muscle index (SMI) indexes, have been proposed for the measurement of muscle mass, yet it remains to be established how they perform in survival prognostication compared to each other in RCC. In previous studies, SMI has been shown to outperform PMI in the evaluation of prognosis [30, 31]. On the other hand, the use of other MIs (such as PMI+ESI) in prognostic evaluation has not been widely studied before. Additionally, although several studies have argued that SMI is superior to PMI in prognostication, there is not much evidence regarding using PMI versus PMI+ESI as a surrogate for SMI when SMI cannot be measured.

This study aimed to compare three commonly used L3-MIs to investigate whether they perform differently in survival prognostication in RCC and whether to use PMI or PMI+ESI when SMI cannot be measured.

## Materials and methods

### Patient sample

We retrospectively enrolled a consecutive cohort of patients with RCC who underwent surgical treatment at Kuopio University Hospital, Finland, between January 1st, 2001 and December 31st, 2015. We included patients with RCC who were ≥ 18 years old and had undergone preoperative CT scans. Muscle mass was evaluated at the L3 level, and we therefore excluded patients who were not imaged with CT preoperatively or who did not have preoperative scans extending to that level. Because height is required for the calculation of MIs, patients with missing height data were also excluded.

The study was approved, and the need for patient consent was waived by the Institutional Review Board of Kuopio University Hospital, Finland. As the study did not change the management of these patients, approval from the Ethics Committee was not needed according to national law.

### Data Collection

The following data were collected from patient records: age at the time of the diagnosis, sex, height, weight, body mass index (BMI), symptoms, comorbidities, and preoperative blood and urine test results. The type of surgery and pathological characteristics of the tumor, such as the TNM stage, WHO/ISUP grade, histology, necrosis, and the number and sites of metastases, were recorded. In addition, information on oncological treatments, such as chemotherapy, targeted agents, and radiotherapy, was collected.

CT images for muscle mass measurements were collected from the hospital’s Picture Archiving and Communications System (PACS). We retrieved the preoperative CT image captured closest to the operation.

### Radiological measurements and muscle and adipose tissue indexes

**Figure 1:**
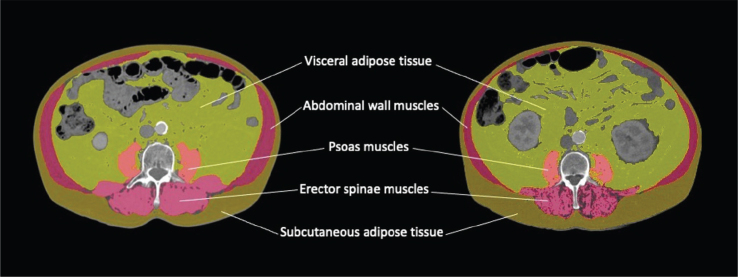
Body composition analyses were performed on axial computed tomography slices at the level of the 3rd lumbar vertebra. The male patient on the left had a normal skeletal muscle index (SMI) value of 55.4 cm^2^/m^2^ (normal ≥ 48.4 cm^2^/m^2^ for males). The male patient on the right had a low SMI value of 41.S cm^2^/m^2^.

One observer performed all muscle measurements with 3DSlicer software [version 4.11.20210226, available at https://www.slicer.org]. Following the pipeline described in [17], the psoas muscle (PM), psoas and erector spinae muscles (PM+ES), and the total skeletal muscle (SM) volumes (cm^3^) were determined from the midpoint of the L3 (Figure 1). The total skeletal muscle area included the bilateral psoas major, erector spinae, quadratus lumborum, transversus abdominis, rectus abdominis, and internal and external abdominal oblique muscles. The Hounsfield unit thresholds were set at -29 to 150 for skeletal muscle tissue. Slice volumes were divided by the slice thicknesses (0.5–5.9 mm) to calculate the corresponding areas (cm^2^). MIs (PMI, PMI+ESI, SMI) were calculated by dividing the corresponding areas by the patient’s squared height.

### Statistical methods

All statistical analyses were performed using SPSS (version 27.0, SPSS Inc., Chicago, IL, USA). *P* values ≤ 0.05 were considered statistically significant. Patient demographics are presented as absolute values and percentages and continuous variables as means and standard deviation (SDs) values unless otherwise stated. We used the Mann-Whitney U test to evaluate the associations between the clinical parameters and continuous variables and the Chi-squared test to test association between nominal variables.

To establish sex-specific cut-off values for low muscle mass, we defined cut-offs using the receiver operating characteristic (ROC) for PMI, PMI+ESI, and SMI according to survival metrics (1-year, 5-year, and OS rates) by calculating the Youden’s Indexes ((sensitivity + specificity) – 1) separately for males and females and chose the MI values with the highest Youden Index as the cut-off. Patients whose muscle mass was equal to or greater than the cut-off value formed the normal muscle mass group and those with muscle mass lower than the cut-off value formed the low muscle mass group. In addition, we determined the sensitivities, specificities**,** and accuracies for muscle mass cut-off values with respect to their ability to predict survival metrics with 95% confidence intervals (CIs). The 95% CIs for sensitivity, specificity, and accuracy were calculated according to Baratloo et al. (2015) [32]. Sensitivity, specificity, and accuracy metrics between the MIs were deemed statistically different when the 95% CIs did not overlap.

Cox regression analyses for survival metrics were performed using both continuous and categorical variables in a uni- and multivariate manner. The total OS was calculated as the number of months elapsed from the date of the surgery until death or the end of the follow-up (December 31st, 2022), whichever occurred earlier. We performed univariate survival analyses with continuous variables separately for males and females because the body composition metrics differed significantly between the sexes. Along with age, BMI, tumor stage, and grade, sex was included in the multivariate analyses when continuous MIs were evaluated. Categorical MIs defined with sex-specific cut-offs were used in the multivariate analysis, and the models were not separately adjusted for sex. In our patient sample, only 28 (13.1%) patients had had a non-clear cell histology. As there were no significant differences between the survival rates of patients with clear cell and non-clear cell histology in univariate models, histopathology was not included in the multivariate model.

## Results

### Sample description

**Figure 2:**
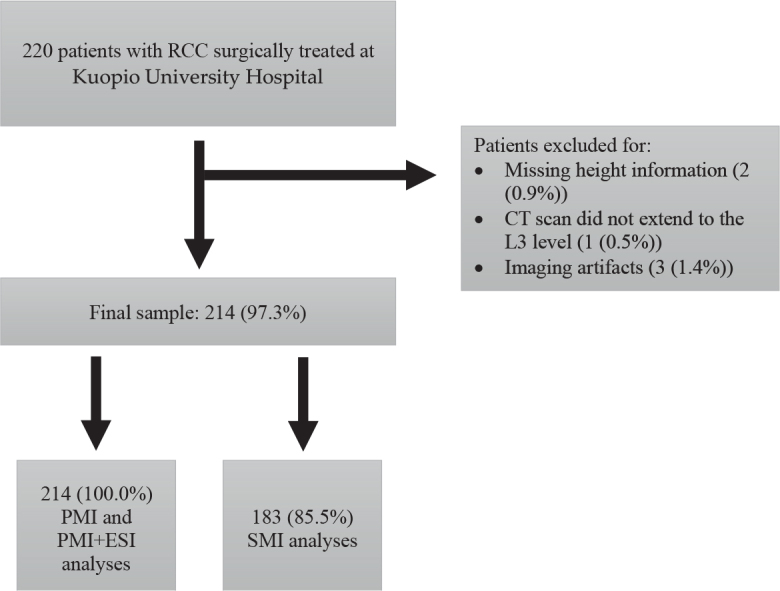
Patient selection flowchart. RCC: renal cell carcinoma, CT: computed tomography, L3: third lumbar vertebra, PMI: psoas muscle index, ESI: erector spinae index, SMI: skeletal muscle index.

Of the 220 patients screened for this study, 214 (65.4 ± 11.9 years) were included in the final study cohort (Figure 2). The final study group consisted of 115 (53.7%) male and 99 (46.3%) female patients. The evaluation of SMI was not possible in 31 (14.5%) patients because the abdominal muscles were not fully in the field of view at the L3 level. PMI and PMI+ESI were evaluable for all the patients in the final study cohort. The patients whose SMI could not be evaluated had a higher BMI in comparison to those whose SMI was evaluable (30.0 ± 6.7 kg/m^2^ vs. 27.6 ± 5.4 kg/m^2^, *P* = 0.015). The median time between the preoperative CT scan and the operation was 33.0 days (range: 1–108 days).

The clinical characteristics of the patients are summarized in Table 1. Male patients were significantly younger than females (63.0 ± 10.9 years vs. 68.3 ± 12.4 years, *P* < 0.001). Clear-cell RCC was the most common histological subtype (*n* = 186, 86.9%). The mean maximum diameter of the tumor measured from surgical resection was 6.3 cm ± 3.7 cm. The majority (*n* = 182, 85.0%) of the patients had a non-metastatic disease at the time of diagnosis; statistically, male and female patients had similar shares of metastatic and non-metastatic diseases (*P* = 0.894). Seven (3.3%) patients had a bilateral disease. Ninety (42.1%) patients died during the mean follow-up period of 7.0 ± 4.0 years, and there were no differences in the occurrence of deaths between males and females (*P* = 0.199).

**Table 1:** Patient characteristics and muscle indexes among male and female patients.

Characteristic	All				Male				Female				*P*
	*n*	%	Mean	SD	*n*	%	Mean	SD	*n*	%	Mean	SD	
Patients	214	100			115	53.7			99	46.3			
Age (years)	214	100	65.4	11.9	115	53.7	63.0	10.9	99	46.2	68.3	12.4	<0.001
BMI^a^ (kg/m^2^)	212	99.1	27.9	5.7	114	53.8	27.7	5.0	98	46.2	28.2	6.4	0.985
WHO/ISUP grade	212	99.1			113	53.3			99	46.7			
Grade 1	34	15.9			13	38.2			21	61.8			
Grade 2	106	49.5			59	55.7			47	44.3			
Grade 3	36	16.8			19	52.8			17	47.2			
Grade 4	36	16.8			22	61.1			14	38.9			
Largest diameter of tumor (cm)	183	85.5	6.3	3.7	95	51.9	6.8	4.2	88	48.1	5.8	3.1	0.134
Metastasis at diagnosis	211	98.6			114	54.0			99	46.0			
Yes	29	13.6			16	55.2			13	44.8			
No	182	85			98	53.8			84	46.2			
Stage	212	99.1			114	53.8			98	46.2			
I	102	47.7			50	49.0			52	51.0			
II	29	13.6			15	51.7			14	48.3			
III	51	23.8			33	64.7			18	35.3			
IV	30	14.0			16	53.3			14	46.7			
Histology	214	100			115	53.7			99	46.2			
Clear cell	186	86.9			95	51.1			91	48.9			
Non-clear cell	28	13.1			20	71.4			8	28.6			
Bilateral disease	213	99.5			114	53.5			99	46.5			
Yes	7	3.3			5	71.4			2	28.6			
No	206	96.3			109	52.9			97	47.1			
Death	214	100			115	53.7			99	46.2			
Yes	90	42.1			53	58.9			37	41.1			
No	124	57.9			62	50.0			62	50.0			
PMI (cm^2^/m^2^)	214	100	6.0	1.7	115	100	6.9	1.6	99	100	5.1	1.3	<0.001
PMI+ESI (cm^2^/m^2^)	214	100	24.6	4.8	115	100	26.5	4.7	99	100	22.4	4.0	<0.001
SMI^b^ (cm^2^/m^2^)	183	85.5	45.7	8.8	95	51.9	50.2	7.9	88	48.1	40.8	7.0	<0.001

The *P* value indicates differences between the sexes.

N: number; SD: standard deviation; BMI: body mass index; PMI: psoas muscle index; ESI: erector spinae index; SMI: skeletal muscle index.

a Reason for missing values: missing information on weight (*n* = 2).

b Reason for missing values: muscle compartment partially out of the field of view.

### Differences in body composition metrics between males and females and sex-specific cut-offs for low and normal MIs

Male patients had higher PMI (6.9 ± 1.6 cm^2^/m^2^ vs. 5.1 ± 1.3 cm^2^/m^2^, *P* < 0.001), PMI+ESI (26.5 ± 4.7 cm^2^/m^2^ vs. 22.4 ± 4.0 cm^2^/m^2^, *P* < 0.001), and SMI (50.2 ± 7.9 cm^2^/m^2^ vs. 40.8 ± 7.0 cm^2^/m^2^, *P* < 0.001) values than female patients. Male and female patients had similar BMIs (males: 27.7 ± 5.0 kg/m^2^ and females: 28.2 ± 6.4 kg/m^2^, *P* = 0.985). Body composition metrics and BMIs of the patients are summarized in Table 1. According to OS, low muscle mass was defined as PMI < 6.4 cm^2^/m^2^, PMI+ESI < 24.9 cm^2^/m^2^, and SMI < 48.4 cm^2^/m^2^ in males and as PMI < 4.8 cm^2^/m^2^, PMI+ESI < 21.2 cm^2^/m^2^, and SMI < 36.3 cm^2^/m^2^ in females (Table 2**).** Using the OS-based cut-offs, the proportion of male vs. female patients with low muscle mass was 70.4% vs. 42.4% (*P* < 0.001; PMI), 37.4% vs. 37.4% (*P* = 0.998; PMI+ESI), and 44.2% vs. 30.7% (*P* = 0.060; SMI).

**Table 2:** Sensitivity, specificity, and accuracy metrics of different MIs using sex-specific cut-off values for 1-year, 5-year, and overall survival.

	All (*n* = 183)							Males (*n* = 95)						Females (*n* = 88)					
	Cut-off (Males / Females) (cm^2^/m^2^)	Sensitivity (%)		Specificity (%)		Accuracy (%)		Sensitivity (%)		Specificity (%)		Accuracy (%)		Sensitivity (%)		Specificity (%)		Accuracy (%)	
		*n*	%	*n*	%	*n*	%	*n*	%	*n*	%	*n*	%	*n*	%	*n*	%	*n*	%
**1-year survival**																			
PMI	7.2 / 3.0	68.4	43.5–87.4	67.1	59.3–74.2	67.2	59.9–74.0	91.7	61.5–99.8	38.6	28.1–49.9	45.3	35.0–55.8	28.6	3.7–71.0	96.3	89.6–99.2	90.9	82.9–96.0
PMI+ESI	24.9 / 16.3	57.9	33.5–79.8	79.9	72.9–85.7	77.6	70.9–83.4	75.0	42.8–94.5	65.1	53.8–75.2	66.3	55.9–75.7	28.6	3.7–71.0	95.1	87.8–98.6	89.8	81.5–95.2
SMI	48.0 / 37.8	73.7	48.8–90.9	64.6	56.8–71.9	65.6	58.2–72.4	83.3	51.6–97.9	66.3	55.1–76.3	68.4	58.1–77.6	57.1	18.4–90.1	63.0	51.5–73.4	62.5	51.5–72.6
**5-year survival**																			
PMI	7.7 / 4.8	81.7	69.6–90.5	52.9	43.6–61.9	62.3	54.9–69.3	91.9	78.1–98.3	39.6	27.1–53.4	60.0	49.4–69.9	65.2	42.7–83.6	64.6	51.8–76.1	64.8	53.9–74.7
PMI+ESI	24.9 / 21.2	63.3	49.9–75.4	71.5	62.7–79.3	68.9	61.6–75.5	59.5	42.1–75.3	72.4	59.1–83.3	67.4	57.0–76.6	69.6	47.1–86.8	70.8	58.2–81.4	70.5	59.8–79.7
SMI	48.4 / 36.3	60.0	46.5–72.4	72.4	63.6–80.0	68.3	61.0–75.0	64.9	47.5–79.8	67.2	53.7–79.0	66.3	55.9–75.7	52.2	30.6–73.2	76.9	64.8–86.47	70.5	59.8–79.7
**Overall survival**																			
PMI	6.4 / 4.8	65.3	53.5–76.0	70.4	60.8–78.8	68.3	61.0–75.0	64.4	48.8–78.1	74.0	59.7–85.4	69.5	59.2–78.5	66.7	47.2–82.71	67.2	53.7–79.0	67.1	56.2–76.7
PMI+ESI	24.9 / 21.2	61.3	49.4–72.4	75.0	65.8–82.8	69.4	62.2–76.0	60.0	44.3–74.3	78.0	64.0–88.5	69.5	59.2–78.5	63.3	43.9–80.1	72.4	59.1–83.3	69.3	58.6–78.7
SMI	48.4 / 36.3	58.7	46.7–69.9	76.9	67.8–84.4	69.4	62.2–76.0	64.4	48.7–78.1	74.0	59.6–85.3	69.5	59.2–78.5	50.0	31.3–68.7	79.3	66.6–88.85	69.3	58.6–78.7

Sensitivity, specificity, and accuracy were determined for patients (*n* = 183) for whom all three MIs (PMI, PMI+ESI and SMI) were available. Sensitivity, specificity, and accuracy metrics were deemed statistically different when the 95% CIs did not overlap.

MI: muscle index; PMI: psoas MI; ESI: erector spinae index; SMI: skeletal MI; CI: confidence interval.

### Survival analyses

Of the 214 patients, 193 (90.2%) were alive one year or more post-surgery and 145 (67.8%) 5 years or more post-surgery. Altogether, 124 (57.9%) patients were alive at the end of the follow-up period. The mean OS times for patients with low vs. normal muscle mass according to PMI, PMI+ESI, and SMI were 70.9 vs. 94.5 months, 65.3 vs. 96.0 months, and 62.2 vs. 96.3 months (all *P* < 0.001), respectively.

Univariable and multivariable models for 1-year survival, 5-year survival, and OS with continuous and categorized MIs are presented in Table 3 and Supplementary Tables S1–S4. Lower PMI, PMI+ESI, and SMI predicted significantly decreased 1-year, 5-year, and OS rates among males in univariate Cox regression analyses (Supplementary Table S1). Among females, PMI, PMI+ESI, and SMI predicted significantly decreased 5-year and OS rates (*P* < 0.05), but not 1-year survival. PMI, PMI+ESI, and SMI values categorized as low according to the cut-offs were significantly associated with all survival parameters (1-year, 5-year, and OS) (Table 3, Supplementary Table S2). The univariate association between clinical characteristics and 1-year, 5-year, and OS rates is reported in Supplementary Tables S1 and S2.

**Table 3:** Uni- and multivariable models for 1-year, 5-year, and overall survival with continuous and categorized muscle indexes. Significant hazard ratios are bolded.

Continous/Categorical	Muscle index	1-year survival		5-year survival		Overall survival	
		Crude^a^	Fully-adjusted^a^	Crude^a^	Fully-adjusted^a^	Crude^a^	Fully-adjusted^a^
		HR (95% CI)	HR (95% CI)	HR (95% CI)	HR (95% CI)	HR (95% CI)	HR (95% CI)
Continous	PMI	**0.76 [0.66–0.88]**	0.72 [0.48–1.06]	**0.73 [0.62–0.87]**	**0.74 [0.60–0.90]**	**0.72 [0.62–0.84]**	**0.74 [0.62–0.89]**
	PMI+ESI	**0.93 [0.89–0.98]**	0.89 [0.78–1.03]	**0.92 [0.87–0.98]**	**0.92 [0.85–0.99]**	**0.92 [0.87–0.96]**	0.94 [0.88–1.01]
	SMI	**0.95 [0.92–0.98]**	**0.90 [0.83–0.99]**	**0.95 [0.92–0.98]**	**0.94 [0.89–0.99]**	**0.94 [0.91–0.97]**	**0.94 [0.90–0.99]**
Categorical	Low PMI^b^	**3.68 [1.48–9.11]**	**3.71 [1.35–10.20]**	**3.09 [1.75–5.48]**	**2.82 [1.55–5.14]**	**2.56 [1.67–3.91]**	**2.52 [1.56–4.08]**
	Low PMI+ESI^c^	**4.90 [2.06–11.62]**	**2.77 [1.13–6.76]**	**2.81 [1.74–4.53]**	**2.29 [1.32–3.96]**	**2.84 [1.87–4.33]**	**2.17 [1.34–3.52]**
	Low SMI^d^	**4.75 [1.71–13.18]**	**5.28 [1.62–17.25]**	**3.10 [1.85–5.20]**	**3.36 [1.83–6.17]**	**3.29 [2.07–5.24]**	**3.14 [1.84–5.38]**

CI: confidence interval; HR: hazard ratio; MI: muscle index; PMI: psoas muscle index; ESI: erector spinae index; SMI: skeletal muscle index.

a Crude continuous values adjusted for sex. Continuous fully-adjusted models adjusted for age, sex, BMI, tumor stage and WHO/ISUP grade. Categorized fully-adjusted models adjusted for age, BMI, tumor stage and WHO/ISUP grade.

b Cut-off values (cm^2^/m^2^) for PMI (males/females): 1-year survival: 7.2/3.0; 5-year survival: 7.7/4.8; overall survival: 6.4/4.8.

c Cut-off values (cm^2^/m^2^) for PMI+ESI (males/females): 1-year survival 24.9/16.3; 5-year survival: 24.9/21.2; overall survival: 24.9/21.2.

d Cut-off values (cm^2^/m^2^) for SMI (males/females): 1-year survival: 48.0/37.8; 5-year survival: 48.4/36.3; overall survival: 48.4/36.3.

Significant hazard ratios are bolded.

In multivariate Cox regression analyses, continuous PMI (HR, 95% CI: 0.74 (0.62–0.89)) and SMI (HR, 95% CI: 0.94 (0.90–0.99)) remained significant predictors of worse OS after the adjustment for sex, stage, and grade (Table 3, Supplementary Table S3). Multivariate Cox regression models indicated that low PMI, PMI+ESI, and SMI were significant predictors of decreased 1-year, 5-year, and OS (Table 3, Supplementary Table S4).

### Comparison between MIs in survival prediction

We compared MIs by evaluating the confidence intervals of their sensitivities, specificities, and accuracies in the prediction of 1-year, 5-year, and OS (Table 2). In males, PMI+ESI (66.3% (95% CI: 55.9%–75.7%)) and SMI (68.4% (95% CI: 58.1%–177.6%)) had better accuracy than PMI (45.3% (95% CI: 35.0%–55.8%)) for the prediction of 1-year survival. In females, PMI (90.9% (95% CI: 82.9%–96.0%)) and PMI+ESI (89.9% (95% CI: 81.5%–95.2%)) had better accuracy than SMI (62.5% (95% CI: 51.5%–72.6%)) for the prediction of 1-year survival. The accuracies of MIs in the prediction of 5-year survival and OS did not differ statistically in either males or females.

## Discussion

In line with other studies, we showed that low muscle mass at the L3 level, defined with sex-specific CT-determined PMI, PMI+ESI, and SMI index cut-off values, is a prognostic factor for worse 1-year, 5-year, and OS in surgically treated patients with RCC. Interestingly, although PMI+ESI and SMI indexes were more accurate than PMI in males and PMI and PMI+ESI indexes more accurate than SMI in females in 1-year survival prediction, we found no differences in accuracy between MIs based on the psoas muscles versus larger muscle areas in long-term survival prediction. When evaluating short-term prognosis, particularly when SMI cannot be evaluated, we promote the use of PMI+ESI because it performed well in both sexes. However, the choice of MI is equivocal for long-term prognostication in RCC.

Several studies have suggested that the MIs computed from muscle areas measured from a single slice at the L3 level are associated with whole-body SMM [10, 33], and it is well established that low muscle mass particularly based on SMI is a prognostic factor in several diseases such as cancer [34]. Interestingly, we found that PMI+ESI and SMI were more accurate than PMI in males and PMI and PMI+ESI more accurate than SMI in females in the prediction of 1-year survival. However, all the indexes performed similarly in 5-year survival and OS prediction. This suggests that PMI+ESI should be used for the prognostication of short-term survival. However, all the MIs perform equally well in long-term prognostication of patients with RCC. We are unaware of studies comparing the three indexes in short- and long-term prognostication, and there are no studies comparing SMI and PMI in patients with RCC either (Table 4). Recently, the use of PMI at the L3 level has attracted strong criticism. Although our results did not suggest inferior performance of PMI in comparison to PMI+ESI or SMI in longer follow-up, several studies have questioned the correlation between L3 PMI and SMI **[**31] and suggested that PMI is unable to accurately classify patients with low and normal muscle mass [30].

**Table 4:** A literature review on cross-sectional imaging-based muscle mass evaluation in surgically treated patients with renal cell carcinoma.

Author (year)	*N* of patients (males/females), age	Locally / locally advanced / metastatic	Subtypes	Description of the patient sample	Imaging modality (level unless L3)	Survival results	Association with clinical factors
Our study	214 (115/99), mean age 65.4 years	131 (62%) / 51 (24%) / 30 (14%)	186 clear cell RCCs, 28 non-clear cell RCCs	Patients undergoing surgical treatment for RCC	CT	Sarcopenia* associated with 1-year (HR 4.8, 95% CI: 1.7–13.2), 5-year (HR 3.1, 95% CI: 1.9–5.2), and overall survival (HR 3.3, 95% CI: 2.1–5.2). * According to SMI cut-offs (males/females): 48.4/36.3 cm^2^/m^2^.	NR
Demirel et al. (2023)	188 (114/74), NR	NR	NR	Patients undergoing partial or radical nephrectomy for RCC	CT	NR	No significant differences between muscle tissue values between patients with and without high-grade Clavien-Dindo complications.
Midenberg et al. (2023)	473 (312/161), mean age 61.7 years	424 (90%) / 49 (10%) / 0 (0%)	392 clear cell RCCs, 81 non-clear cell RCCs	Patients undergoing partial or radical nephrectomy for localized RCC	CT, MRI	Sarcopenia* associated with shorter OS (HR 1.51, 95% CI: 1.07–2.13) but not RFS (HR 1.33, 95% CI: 0.88–2.03) or CSS (HR 1.66, 95% CI: 0.96–2.87). * For patients with a BMI <30 kg/m^2^, sarcopenia was defined as SMI <47/<38 cm^2^/m^2^ (males/females). For patients with a BMI≥30 kg/m^2^, sarcopenia was defined as SMI <54/<47 cm^2^/m^2^ (males/females).	NR
Khan et al. (2022)	158 (120/38), mean age 61.3 years	0 (0%) / 0 (0%) / 158 (100%)	117 clear cell RCCs, 41 non-clear cell RCCs	Patients undergoing cytoreductive nephrectomy for mRCC	CT, MRI	The median OS was 15.0 months (95% CI: 9.3–26.4) for sarcopenic* patients and 29.4 months (95% CI: 20.0–54.6) for nonsarcopenic patients (*P* = 0.040). * According to SMI cut-off <43 cm^2^/m^2^ for males with BMI <25, <53 cm^2^/m^2^ for males with BMI≥25, and <41 cm^2^/m^2^ for all females.	NR
Maurits et al. (2022)	1,039 (643/396), NR	545 (52%) / 174 (17%) / 320 (31%)	655 clear cell RCCs, 384 non-clear cell RCCs or RCCs of unknown type	Patients with stage I–IV RCC	CT	Stages I–III: For females, a 10-unit increase in SMI was associated with worse RFS (HR 2.01, 95% CI: 1.17–3.45) while no association was observed for males (HR 0.97, 95% CI: 0.72–1.30). Stage IV: No significant associations between SMI and OS were observed.	NR
Lee et al. (2021)	632 (NR), NR	632 (100%) / 0 (0%) / 0 (0%)	NR	Patients undergoing radical nephrectomy for stage I–II RCC	CT	Sarcopenia* was associated with worse overall survival (94.0% versus 82.1%, *P* < 0.001). In the multivariate analysis, sarcopenia was an independent risk factor for all-cause mortality (HR 2.58, 95% CI: 1.02–6.54) and cancer-specific mortality (HR 3.07, 95% CI: 1.38–6.83). * According to SMI cut-offs (males/females): 52.4/38.5 cm^2^/m^2^.	NR
Higgins et al. (2021)	352 (236/116), mean age 62.7 years	296 (84%) / 56 (16%) / 0 (0%)	NR	Patients undergoing partial or radical nephrectomy for localized RCC	CT, MRI	On multivariate analysis, sarcopenia* was an independent predictor of worse OS (HR 1.64, 95% CI: 1.15–2.34, *P* = 0.006) and CSS (HR 2.01, 95% CI: 1.19–3.39, *P* = 0.009). * According to SMI cut-offs of <47 cm^2^/m^2^ for males and <38 cm^2^/m^2^ for females with BMI <30 and <54 cm^2^/m^2^ for males and <47 cm^2^/m^2^ for females with BMI≥30.	NR
Watanabe et al. (2021)	83 (63/20), NR	0 (0%) / 49 (59%) / 34 (41%)	70 clear cell RCCs, 13 non-clear cell RCCs or RCCs of unknown type	Patients with RCC with inferior vena cava thrombus undergoing nephrectomy and thrombectomy	CT, MRI	The median OS was 32.0 months (95% CI: 16.5-NR) for sarcopenic* patients. * According to SMI cut-offs (males/females): 52.4/38.5 cm^2^/m^2^.	No significant differences in the incidence rates of surgical complications or the duration of hospitalization after surgery between sarcopenic and non-sarcopenic patients.
Darbas et al. (2020)	96 (68/28), median age 66 years	96 (100%) / 0 (0%) / 0 (0%)	81 clear cell RCCs, 9 papillary RCCs, 6 chromophobe RCCs	Overweight or obese (as assessed by BMI at diagnosis) patients undergoing partial or radical nephrectomy with localized RCC without adjuvant treatment	CT	In univariate analyses, sarcopenia* was associated with OS (HR 0.4, 95% CI: 0.1–0.9). In multivariate analyses, sarcopenia was not an independent prognostic factor for OS (HR 0.5, 95% CI: 0.2–1.4). * According to SMI cut-offs (males/females): 53/41 cm^2^/m^2^.	No significant differences in the occurrence of infections were observed.
Noguchi et al. (2020)	316 (316/0), mean age 63 years	255 (81%) / 61 (19%) / 0 (0%)	Clear cell RCC	Male patients undergoing partial or radical nephrectomy with localized clear cell RCC	CT (L4 level)	The OS and CSS rates in the lower and higher PMI* groups did not differ (*P* = 0.482 and *P* = 0.367, respectively). A lower PMI was a significant predictor of 5-year RFS (*P* = 0.022, HR 2.306). * According to PMI cut-off 409.64 mm^2^/m^2^.	NR
Psutka et al. (2016)	387 (254/133), mean age 65 years	161 (42%) / 221 (57%) / 5 (1%)	316 clear cell RCCs, 35 papillary RCCs, 23 chromophobe RCCs, 4 clear cell papillary RCCs, 1 collecting duct RCC, 8 not otherwise specified RCCs	Patients undergoing radical nephrectomy for localized RCC	CT	Sarcopenic* patients had inferior 5-year CSS (79% vs. 85%, *P* = 0.050) compared to non-sarcopenic patients, and worse 5-year OS (65% vs. 74%, *P* = 0.005). On multivariable analysis sarcopenia was associated with increased cancer specific mortality (HR 1.70, *P* = 0.047) and all-cause mortality (HR 1.48, *P* = 0.039). * According to SMI cut-offs (males/females): 55/39 cm^2^/m^2^.	NR
Fukushima et al. (2016)	37 (32/5), NR	0 (0%) / 0 (0%) / 37 (100%)	34 clear cell RCCs, 3 non-clear cell RCCs or RCCs of unknown type	Patients undergoing cytoreductive nephrectomy for mRCC	CT	ΔSMI* was significantly associated with OS (HR 0.920, *P* < 0.001). * SMI was calculated ≤1 month before and 5 to 6 months after cytoreductive nephrectomy. ΔSMI was calculated as follows: [(postoperative SMI − preoperative SMI) /preoperative SMI] × 100.	NR
Peyton et al. (2016)	128 (85/43), mean age 63 years	7 (5%) / 87 (68%) / 34 (27%)	99 clear cell RCCs, 31 non-clear cell RCCs	Patients undergoing radical nephrectomy for stage III–IV RCC	CT, MRI	Sarcopenia* was not associated with OS (*P* = 0.210). * Determined as the lowest sex specific psoas muscle quartile.	Sarcopenia was associated with a risk of Clavien grade ≥III complication (*P* = 0.030) and node-positive disease (*P* = 0.010).
Sharma at al. (2015)	93 (63/28), median age 61 years	0 (0%) / 0 (0%) / 93 (100%)	70 clear cell RCCs, 23 non-clear cell RCCs	Patients undergoing cytoreductive nephrectomy for mRCC	CT, MRI	Median OS in sarcopenic* patients was 7 months (95% CI: 0.8–13.2) vs. 23 months (95% CI: 12.4–33.6) in nonsarcopenic patients. In the multivariate analysis, sarcopenia was an independent predictor of OS (HR 2.13, 95% CI: 1.15–3.92, *P* = 0.016). * According to SMI cut-off <43 cm^2^/m^2^ for males with BMI <25, <53 cm^2^/m^2^ for males with BMI≥25, and <41 cm^2^/m^2^ for all females.	Sarcopenic patients received neoadjuvant systemic therapy more often (*P* = 0.022).

N: number; RCC: renal cell carcinoma; CT: computed tomography; HR: hazard ratio; CI: confidence interval; SMI: skeletal muscle index; mRCC: metastatic RCC; OS: overall survival; PFS: progression-free survival; RFS: recurrence-free survival; CSS: “cancer-specific survival; BMI: body mass index; ICB: immune-checkpoint blockade; PMI: psoas muscle index; SMM: skeletal muscle mass; DLT: dose-limiting toxicity; SMD: skeletal muscle density.

In our study cohort, 14.5% of patients did not qualify for SMI evaluation because their abdominal muscles were out of the field of view. Notably, high BMI is a risk factor for not being suitable for SMI evaluation. The choice of MI when SMI is not evaluable remains an unanswered question. It is likely that the number of patients not suitable for SMI evaluation reflects the number of obese patients. In populations with higher rates of obesity, SMI not being evaluable may be more often encountered, whereas the scenario may not be as common in leaner populations. As both obesity and the use of MRI (often with narrower bore) increase [35, 36], alternatives to SMI are of interest particularly to these patients. PMI and PMI+ESI were evaluable for all the patients in the cohort. Given that PMI+ESI performed well in both sexes in short-term prognostication in addition to being statistically on par with PMI in long-term prognostication, we promote the use of PMI+ESI over PMI when SMI is not evaluable.

Our study has some limitations to note. For example, this retrospective study is unable to answer why the three indexes perform differently in short-term prognostication. Furthermore, as there are no other studies directly comparing the three indexes, we advocate further research to study whether our findings regarding the different performances of MIs can be replicated in other diseases.

## Conclusions

Low imaging-based muscle mass, defined by PMI, PMI+ESI, and SMI, is a marker of impaired survival. PMI+ESI performed well overall in both sexes in short-term prognostication, but there were no differences between PMI, PMI+ESI, and SMI in long-term prognostication. Particularly when SMI cannot be evaluated, we recommend using PMI+ESI for prognostication.

## Supplementary Material



## Data Availability

The data that supports the findings of this study is available from registry authorities. Researchers are not allowed to forward confidential non-anonymous health data to any third parties. All the relevant data is presented in the manuscript.
