# Supplementary material for: Comparison of different 2D muscle indexes measured at the level of the 3rd lumbar vertebra in survival prediction in patients with renal cell carcinoma
Source: Acta Oncol. 2024 May 14;63:27450. doi: 10.2340/1651-226X.2024.27450 (PMC11332504; doi:10.2340/1651-226X.2024.27450)
Supplement: Supplementary file 1 [file AO-63-27450-s1.pdf]

Supplementary material has been published as submitted. It has not been copyedited, or typeset by Acta Oncologica

Supplementary table 1. Univariate models for 1-year, 5-year, and overall survival for the whole cohort and males and females separately. Muscle indexes are used as continuous variables.

	All			Males			Females		
	1-year survival (HR [95% CI])	5-year survival (HR [95% CI])	Overall survival (HR [95% CI])	1-year survival (HR [95% CI])	5-year survival (HR [95% CI])	Overall survival (HR [95% CI])	1-year survival (HR [95% CI])	5-year survival (HR [95% CI])	Overall survival (HR [95% CI])
Age (years)	**1.03 [1.01-1.05]**	**1.03 [1.01-1.05]**	**1.03 [1.01-1.05]**	**1.03 [1.01-1.06]**	**1.04 [1.01-1.07]**	**1.04 [1.02-1.07]**	1.03 [1.00-1.06]	1.03 [1.00-1.06]	1.03 [1.00-1.07]
WHO/ISUP grade									
Grade 1	REF	REF	REF	REF	REF	REF	REF	REF	REF
Grade 2	>100* [0.00->100]	2.84 [0.86-9.40]	**2.63 [1.03-6.70]**	>100* [0.00->100]	1.82 [0.42-7.92]	2.53 [0.59-10.77]	>100* [0.00->100]	4.28 [0.54-33.76]	2.48 [0.71-8.64]
Grade 3	>100* [0.00->100]	**5.76 [1.66-20.06]**	**4.44 [1.65-11.98]**	>100* [0.00->100]	2.92 [0.61-14.06]	3.91 [0.85-17.97]	>100* [0.00->100]	**11.66 [1.43-94.84]**	**4.65 [1.23-17.57]**
Grade 4	>100* [0.00->100]	**14.78 [4.47-48.86]**	**12.21 [4.72-31.55]**	>100* [0.00->100]	**8.09 [1.86-35.12]**	**10.62 [2.46-45.84]**	>100* [0.00->100]	**27.57 [3.51->100*]**	**12.07 [3.38-43.08]**
Histology									
Clear cell	REF	REF	REF	REF	REF	REF	REF	REF	REF
Non-clear cell	0.92 [0.22-3.12]	1.16 [0.56-2.43]	1.25 [0.63-2.50]	1.18 [0.26-5.34]	1.03 [0.46-2.33]	1.09 [0.51-2.32]	0.62 [0.08-5.07]	2.40 [0.33-17.67]	3.20 [0.44-23.38]
Stage									
Stage 1	REF	REF	REF	REF	REF	REF	REF	REF	REF
Stage 2	**10.59 [1.10->100*]**	**2.83 [1.31-6.09]**	**2.68 [1.43-4.99]**	**10.11 [1.05-97.18]**	**3.52 [1.38-8.93]**	**2.90 [1.25-6.71]**	1.00 [0.00->100*]	1.96 [0.49-7.85]	2.38 [0.94-6.05]
Stage 3	4.13 [0.37-45.50]	**2.21 [1.11-4.42]**	**1.93 [1.08-3.43]**	3.16 [0.29-34.87]	1.80 [0.76-4.23]	1.87 [0.91-3.85]	1.00 [0.00->100*]	2.77 [0.84-9.07]	1.74 [0.61-4.99]
Stage 4	**67.32 [8.87->100*]**	**12.25 [6.52-23.04]**	**9.66 [5.56-16.78]**	**27.37 [3.36->100*]**	**7.63 [3.33-17.51]**	**6.76 [3.19-14.32]**	0.74 [0.00->100*]	**21.06 [7.77-57.06]**	**12.77 [5.64-28.89]**
Body mass index (kg/m^2)	**0.93 [0.89-0.97]**	**0.92 [0.88-0.96]**	**0.92 [0.88-0.96]**	**0.94 [0.89-0.99]**	**0.93 [0.88-0.99]**	**0.93 [0.87-0.99]**	**0.93 [0.87-0.99]**	**0.91 [0.85-0.97]**	**0.91 [0.85-0.97]**
PMI (cm^2/m^2)	**0.85 [0.75- 0.97]**	**0.83 [0.73-0.94]**	**0.82 [0.72- 0.93]**	**0.75 [0.63-0.90]**	**0.72 [0.60-0.86]**	**0.72 [0.60-0.86]**	0.78 [0.60-1.02]	**0.75 [0.58-0.98]**	**0.73 [0.56-0.96]**
PMI+ESI (cm^2/m^2)	0.94 [0.86-1.03]	**0.95 [0.90-0.99]**	**0.94 [0.90-0.99]**	**0.88 [0.77-0.99]**	**0.93 [0.87-1.00]**	**0.93 [0.87-0.98]**	0.98 [0.83-1.17]	**0.90 [0.82-0.99]**	**0.90 [0.83-0.98]**
SMI (cm^2/m^2)	0.98 [0.95- 1.00]	**0.97 [0.95-1.00]**	**0.97 [0.95-1.00]**	**0.95 [0.91-0.99]**	**0.95 [0.91-0.98]**	**0.94 [0.91-0.98]**	0.95 [0.90-1.00]	**0.94 [0.89-0.99]**	**0.94 [0.89-0.99]**

Abbreviations. HR: hazard ratio, CI: confidence interval, PMI: psoas muscle index, ESI: erector spinae index, SMI: skeletal muscle index.
* >100 denotes that the HR or the upper CI are high values as a result small statistical power.

Supplementary table 2. Univariate models for 1-year, 5-year, and overall survival with categorized muscle indexes.

	1-year survival (HR [95% CI])	5-year survival (HR [95% CI])	Overall survival (HR [95% CI])
Age (years)	**1.03 [1.01- 1.05]**	**1.03 [1.01- 1.05]**	**1.03 [1.01- 1.05]**
Sex			
Female	REF	REF	REF
Male	1.43 [0.59-3.44]	1.38 [0.85-2.23]	1.27 [0.84-1.94]
BMI (kg/m^2)			
≥25	REF	REF	REF
<25	1.26 [0.51-3.13]	**1.65 [1.02-2.67]**	**1.65 [1.02-2.67]**
WHO/ISUP grade			
Grade 1	REF	REF	REF
Grade 2	>100* [0.00->100]	2.84 [0.86-9.40]	**2.63 [1.03-6.70]**
Grade 3	>100* [0.00->100]	**5.76 [1.66-20.06]**	**4.44 [1.65-11.98]**
Grade 4	>100* [0.00->100]	**14.78 [4.47-48.86]**	**12.21 [4.72- 31.55]**
Stage			
Stage 1	REF	REF	REF
Stage 2	**10.59 [1.10->100*]**	**2.83 [1.31-6.09]**	**2.68 [1.43-4.99]**
Stage 3	4.13 [0.37-45.50]	**2.21 [1.12-4.42]**	**1.93 [1.082-3.425]**
Stage 4	**67.32 [8.87->100*]**	**12.25 [6.52-23.04]**	**9.66 [5.56-16.78]**
Muscle mass (PMI)			
Normal	REF	REF	REF
Low	**3.68 [1.48-9.11]**	**3.09 [1.75-5.48]**	**2.56 [1.67-3.91]**
Muscle mass (PMI+ESI)			
Normal	REF	REF	REF
Low	**4.90 [2.06-11.62]**	**2.81 [1.74-4.53]**	**2.84 [1.87-4.33]**
Muscle mass (SMI)			
Normal	REF	REF	REF
Low	**4.75 [1.71-13.18]**	**3.10 [1.85-5.20]**	**3.29 [2.07-5.24]**

Abbreviations. HR: hazard ratio, CI: confidence interval, PMI: psoas muscle index, ESI: erector spinae index, SMI: skeletal muscle index, BMI: body mass index.

Cut-off values (cm^2/m^2) for males/females: 1-year PMI: 7.2/3.0; 5-year PMI: 7.7/4.8; OS PMI: 6.4/4.8, 1-year PMI+ESI: 24.9/16.3; 5-year PMI+ESI: 24.9/21.2; OS PMI+ESI: 24.9/21.2, and 1-year SMI: 48.0/37.8; 5-year SMI: 48.4/36.3; OS SMI: 48.4/36.3.

* >100 denotes that the HR or the upper CI are high values as a result small statistical power.

Supplementary table 3. Multivariate models for 1-year, 5-year, and overall survival adjusted for continuous muscle indexes, age, sex, stage, grade, and BMI.

	1-year survival			5-year survival			Overall survival		
	PMI HR [95% CI]	PMI+ESI HR [95% CI]	SMI HR [95% CI]	PMI HR [95% CI]	PMI+ESI HR [95% CI]	SMI HR [95% CI]	PMI HR [95% CI]	PMI+ESI HR [95% CI]	SMI HR [95% CI]
Age (years)	1.03 [0.98-1.08]	1.01 [0.95-1.06]	0.99 [0.93-1.04]	1.02 [0.99-1.04]	1.01 [0.98-1.03]	1.01 [0.98-1.04]	**1.03 [1.01-1.06]**	1.02 [0.99-1.05]	1.02 [0.99-1.05]
Sex									
Female	REF	REF	REF	REF	REF	REF	REF	REF	REF
Male	2.70 [0.74-9.82]	2.13 [0.68-6.70]	**3.35 [1.02-11.81]**	**1.94 [1.01-3.74]**	1.46 [0.79-2.69]	2.17 [1.06-4.47]	**2.12 [1.19-3.76]**	1.48 [0.86-2.55]	**2.36 [1.22-4.56]**
Stage									
Stage 1	REF	REF	REF	REF	REF	REF	REF	REF	REF
Stage 2	6.06 [0.61-60.09]	5.43 [0.54-54.53]	2.67 [0.23-31.68]	1.91 [0.86-4.25]	1.83 [0.82-4.07]	1.31 [0.51-3.33]	1.71 [0.88-3.30]	1.65 [0.86-3.19]	1.05 [0.47-2.32]
Stage 3	2.03 [0.18-22.91]	1.89 [0.16-22.04]	2.01 [0.18-23.00]	1.40 [0.67-2.93]	1.44 [0.69-3.04]	1.42 [0.64-3.12]	1.19 [0.64-2.20]	1.27 [0.68-2.37]	1.13 [0.57-2.22]
Stage 4	**37.22 [4.42->100*]**	**29.23 [3.58->100*]**	**23.34 [2.82->100*]**	**7.99 [3.91-16.33]**	**6.82 [3.37-13.78]**	**7.71 [3.50-16.96]**	**7.03 [3.72-13.29]**	**5.80 [3.11-10.80]**	**5.99 [2.90-12.34]**
WHO/ISUP grade									
Grade 1	REF	REF	REF	REF	REF	REF	REF	REF	REF
Grade 2	>100* [0.00->100]	>100* [0.00->100]	>100* [0.00->100]	1.73 [0.51-5.91]	1.78 [0.52-6.04]	1.82 [0.41-8.06]	1.76 [0.67-4.59]	1.81 [0.70-4.72]	2.13 [0.63-7.18]
Grade 3	>100* [0.00->100]	>100* [0.00->100]	>100* [0.00->100]	**3.72 [1.01-13.70]**	3.68 [0.99-13.62]	**5.19 [1.10-24.57]**	**2.90 [1.01-8.31]**	**2.93 [1.03-8.36]**	**4.58 [1.25-16.80]**
Grade 4	>100* [0.00->100]	>100* [0.00->100]	>100* [0.00->100]	**4.39 [1.21-15.98]**	**5.17 [1.43-18.77]**	**5.65 [1.20-26.56]**	**4.09 [1.46-11.45]**	**4.59 [1.65-12.80]**	**6.06 [1.68-21.83]**
BMI (kg/m^2)	1.03 [0.92-1.16]	1.01 [0.91-1.13]	1.04 [0.92-1.18]	1.00 [0.95-1.06]	0.99 [0.93-1.04]	1.01 [0.94-1.08]	0.99 [0.94-1.05]	0.98 [0.93-1.03]	0.99 [0.93-1.06]
MI (cm^2/m^2)	0.72 [0.48-1.06]	0.89 [0.78-1.03]	**0.90 [0.83-0.99]**	**0.74 [0.60-0.90]**	**0.92 [0.85-0.99]**	**0.94 [0.89-0.99]**	**0.74 [0.62-0.89]**	0.94 [0.88-1.01]	**0.94 [0.90-0.99]**

Abbreviations. HR: hazard ratio, CI: confidence interval, MI: muscle index, PMI: psoas muscle index, ESI: erector spinae index, SMI: skeletal muscle index, BMI: body mass index.

* >100 denotes that the HR or the upper CI are high values as a result small statistical power.

Supplementary table 4. Multivariate models for 1-year, 5-year, and overall survival adjusted with categorized muscle indexes, age, BMI, stage, and grade.

	1-year survival			5-year survival			Overall survival		
	PMI HR [95% CI]	PMI+ESI HR [95% CI]	SMI HR [95% CI]	PMI HR [95% CI]	PMI+ESI HR [95% CI]	SMI HR [95% CI]	PMI HR [95% CI]	PMI+ESI HR [95% CI]	SMI HR [95% CI]
Age (years)	1.04 [0.98-1.09]	1.01 [0.97-1.06]	0.99 [0.94-1.05]	1.02 [0.99-1.04]	1.00 [0.98-1.03]	1.00 [0.98-1.03]	**1.02 [1.00-1.05]**	1.02 [0.99-1.04]	1.02 [0.99-1.04]
BMI									
≥ 25	REF	REF	REF	REF	REF	REF	REF	REF	REF
< 25	0.77 [0.29-2.05]	0.78 [0.31-2.01]	0.56 [0.20-1.63]	0.88 [0.52-1.48]	0.89 [0.53-1.51]	0.79 [0.44-1.43]	0.79 [0.49-1.27]	0.88 [0.55-1.40]	0.82 [0.49-1.39]
Stage									
Stage 1	REF	REF	REF	REF	REF	REF	REF	REF	REF
Stage 2	6.17 [0.63-60.76]	5.27 [0.52-53.63]	2.76 [0.23-33.64]	1.89 [0.86-4.17]	1.83 [0.82-4.08]	1.34 [0.52-3.47]	1.66 [0.86-3.21]	1.62 [0.83-3.12]	1.04 [0.46-2.34]
Stage 3	1.87 [0.17-21.14]	1.95 [0.17-22.34]	2.10 [0.19-23.82]	1.47 [0.72-3.00]	1.42 [0.69-2.93]	1.35 [0.62-2.93]	1.34 [0.74-2.42]	1.27 [0.70-2.33]	1.10 [0.57-2.12]
Stage 4	**35.90 [4.20->100*]**	**26.71 [3.19->100*]**	**23.57 [2.67->100*]**	**7.89 [3.87-16.10]**	**6.27 [3.12-12.61]**	**8.97 [3.94-20.43]**	**7.91 [4.21-14.88]**	**5.46 [2.96-10.04]**	**7.18 [3.38-15.27]**
WHO/ISUP grade									
Grade 1	REF	REF	REF	REF	REF	REF	REF	REF	REF
Grade 2	>100* [0.00->100]	>100* [0.00->100]	>100* [0.00->100]	1.74 [0.52-5.88]	1.90 [0.57-6.40]	1.77 [0.40-7.80]	1.93 [0.75-4.98]	1.90 [0.74-4.92]	2.08 [0.62-6.97]
Grade 3	>100* [0.00->100]	>100* [0.00->100]	>100* [0.00->100]	3.44 [0.95-12.46]	**4.03 [1.11-14.63]**	**5.42 [1.15-25.47]**	**3.35 [1.20-9.39]**	**3.32 [1.18-9.33]**	**5.02 [1.37-18.37)**
Grade 4	>100* [0.00->100]	>100* [0.00->100]	>100* [0.00->100]	**4.53 [1.29-15.87]**	**5.78 [1.66-20.09]**	**4.75 [1.01-22.23]**	**4.78 [1.77-12.91]**	**5.56 [2.07-14.98]**	**5.69 [1.58-20.47]**
Muscle mass									
Normal	REF	REF	REF	REF	REF	REF	REF	REF	REF
Low	**3.71 [1.35-10.20]**	**2.77 [1.13-6.76]**	**5.28 [1.62-17.25]**	**2.82 [1.55-5.14]**	**2.29 [1.32-3.96]**	**3.36 [1.83-6.17]**	**2.52 [1.56-4.08]**	**2.17 [1.34-3.52]**	**3.14 [1.84-5.38]**

Abbreviations. HR: hazard ratio, CI: confidence interval, PMI: psoas muscle index, ESI: erector spinae index, SMI: skeletal muscle index.

Cut-off values (cm^2/m^2) for males/females: 1-year PMI: 7.2/3.0; 5-year PMI: 7.7/4.8; OS PMI: 6.4/4.8, 1-year PMI+ESI: 24.9/16.3; 5-year PMI+ESI: 24.9/21.2; OS PMI+ESI: 24.9/21.2, and 1-year SMI: 48.0/37.8; 5-year SMI: 48.4/36.3; OS SMI: 48.4/36.3.

* >100 denotes that the HR or the upper CI are high values as a result small statistical power.
